# Remarkably preserved cysts of the extinct synurophyte, *Mallomonas ampla*, uncovered from a 48 Ma freshwater Eocene lake

**DOI:** 10.1038/s41598-020-61993-1

**Published:** 2020-03-23

**Authors:** Peter A. Siver

**Affiliations:** Department of Botany, Connecticut College, New London, CT 06320 USA

**Keywords:** Palaeontology, Freshwater ecology, Palaeoecology

## Abstract

Chrysophyte algae produce a siliceous stage in their life cycle, through either asexual or sexual reproduction, known as a cyst. Cysts form in response to shifts in environmental conditions, population density, or predation pressure, and upon germination provide a seed source for future populations. Cysts are morphologically distinct for each species, and since their remains become part of the sediment or fossil record cysts are valuable tools in ecological and paleolimnological investigations. However, their value as biological indicators is limited because the vast majority of cyst morphotypes have not been linked to specific vegetative species. In the current work, an exquisitely preserved and morphologically complex cyst type is described from a 48 million year old early Eocene fossil site. This finding is remarkable since many of the cysts were still associated with components of the living vegetative cells that produced them, enabling the morphotype to be immediately linked to the synurophyte, *Mallomonas ampla*. Fusion of identifiable components of the living cell post cyst formation is unknown in modern investigations. The identification of the cyst structure for *M*. *ampla* could be valuable in determining cyst morphotypes for other species in the lineage.

## Introduction

The Chrysophyceae, commonly referred to as golden-brown algae, is a diverse, cosmopolitan, and ecologically significant group of heterokont algae that is especially important in freshwater ecosystems^1–4^. Species are mostly microscopic, planktonic or attached, autotrophic, heterotrophic or mixotrophic, naked or with a cell covering, motile or non-motile, and the class embraces numerous vegetative forms^3,5^. Synurophytes are a monophyletic clade of chrysophytes that construct a highly organized covering around the cell composed of distinctive siliceous scales^3^.

As is true with all members of the Chrysophyceae^6,7^, synurophytes are capable of forming a siliceous stage known as a stomatocyst, statospore, or more commonly a cyst, that serves as a resting stage in the life cycle of the species^3,8^. Cysts are presumably produced as a result of either asexual or sexual reproduction^1,4,9^, and their formation is often triggered by sudden changes in environmental conditions, predation pressure^6,10^, or in the case of sexually-produced cysts, population density^4^. Cysts form a seed bank and when conditions once again become favorable for growth, they germinate to initiate a new population.

Cysts are hollow structures, often more or less spherical in shape, with a single germination pore, that are formed endogenously within a silica deposition vesicle (SDV)^1,3^. The SDV takes the shape of the cyst, enclosing a large percentage of the cell cytoplasm, including the nucleus and other vital organelles. The cyst wall forms within the SDV in what is thought to be a two-step process^11,12^. The first step involves the deposition of an inner wall, which is unornamented, morphologically similar for many species, and often different in appearance from the mature cyst^3,11^. Additional outer wall layers comprise the second step of cyst formation. In some cases, the final layer simply results in a smooth outer surface. In other cases, additional wall ornamentation is added and, if present, a collar constructed. The collar is a specialized structure that surrounds the cyst pore, the latter of which becomes plugged with an organic material during the final stages of cyst maturation.

Details describing cyst morphology are given in Duff *et al*.^8^ and Wilkinson *et al*.^13^. Briefly, cysts have anterior and posterior hemispheres, divided by an equator, with the pore usually being situated atop of the anterior hemisphere. Although the majority of cyst types are spherical, there is a wide diversity of shapes, including oval, ovate, oblong, as well as flattened pancake forms. Pore openings are almost always circular, but the inner sides can be straight, conical or concave. Pores may be simple or surrounded by a thick and continuous rim of silica called the collar. Collars may be simple or complex, the latter consisting of two or more separate collars surrounding the pore in a concentric fashion. The outer wall of the cyst can be smooth, or consist of a multitude of different types of elements, including papillae, nodules, spines, ridges, reticulation designs and depressions. The ornamentation of the mature outer wall and the pore-collar complex is of taxonomic importance^6,8,11–13^.

Since the morphology of a mature cyst is specific for a given species, the remains of a cyst type can indicate the presence of that species in a given waterbody. Since many chrysophyte species are found under specific environmental conditions^3,5^, the remains of their cysts can serve as valuable paleoindicators^9^. Cysts have been used to reconstruct a range of environmental parameters, including nutrient conditions^14^, pH^15–17^, specific conductivity^18,19^ and climate^7,20,21^. Since cysts are also found in the fossil record as far back as the Late Triassic^22^, they also represent potential proxies for reconstructing the geologic past. Hundreds of cyst morphotypes are known, but the vast majority have not been linked to specific clades or actual species^1,6^, inhibiting their full use as bioindicators.

The fossil synurophyte species *Mallomonas ampla* Siver & Lott was described from an early Eocene Arctic deposit, the Giraffe Pipe locality^23^. Although this taxon is extinct, a closely related modern species, *Mallomonas neoampla* Gusev & Siver, was recently described from tropical Vietnam^24^, and both of these taxa are closely related to another modern species, *M*. *multisetigera* Dürrschmidt^25^. The cyst is not known for either of the living species. Recently, numerous cysts still bearing scales belonging to *M*. *ampla* were uncovered from the Giraffe Pipe fossil locality. The purpose of this study is to document and describe the cyst produced by *M*. *ampla*, and to discuss the remarkable and unique conditions under which these fossils were formed.

## Site Description

Details of the Giraffe Pipe fossil locality are given in previous works e.g.^26–28^ and briefly summarized here. The Giraffe Pipe locality is a kimberlite diatreme that was emplaced into the Slave Craton in the Northwest Territories of Canada (64°44′N, 109°45′W) approximately 48 million years ago during the Eocene. The diatreme crater filled with water, becoming a maar lake, and subsequently infilled over thousands of years with a sequence of lacustrine and eventually paludal sediments. The sediment strata were later capped by Neogene glacial deposits, entombing them within the crater. A 163 m long drilled core, collared at a 47° angle, was uncovered from the Giraffe maar in 1999 by BHP Billiton Inc. A 113.1 m portion of the core contains well preserved stratified organic sediment, including 68.3 m of lacustrine mudstones, overlain with 44.8 m of peaty and terrestrial remains. An air-fall tephra bed located near the end of the aquatic phase indicates that the lake sediments were all deposited during the Eocene^28^.

This region of the Arctic was warm and wet compared to today, with reconstructed mean annual temperature and mean annual precipitation values 17 °C higher and 4 times greater, respectively, than present, and the region supported a warm mixed forest^28^. The entire lacustrine phase of the core contains an extensive array of microfossils which reflect a lake teaming with organisms, including chrysophytes, synurophytes, diatoms, euglyphids, heliozoans and sponges^26,29–33^. Shifts in the complement of organisms indicate changing lake conditions, including between alkaline and acidic periods, and shallow versus deep phases^32^. The presence of synurophytes, diatoms and sponge lineages found today largely in tropical regions reflect the warm climate of the region^26^. In addition, the remains of palm trees indicate ice-free conditions and winter temperatures above freezing^34^. The current hypothesis is that post phreatomagmatic kimberlite emplacement, a waterbody formed within the crater, varying in depth and physical attributes over time, slowly infilling, and eventually transitioning to a terrestrial environment.

## Materials and Methods

Samples for this study were obtained from seven sections of a drilled core taken from the Giraffe Pipe locality. Samples from this core are identified using a three-part number^29^. The first number represents the core box in which the sample is stored. Core boxes are numbered sequentially starting with box 1 (closest to the land surface), and increasing with depth in the core. The larger the number, the deeper the section is within the core. Box 11 represents the top and end of the lacustrine phase within the sequence. Each box contains three 1.5 m core lengths, stored in channels 1, 2 and 3. The second number represents the channel. The third number is the measurement in cm down from the top of a core length within the channel. For example, sample 19-1-100 represents a sample taken from 100 cm down along the core length positioned in channel 1 from box 19. This study includes samples from 17-1-15, 17-2-40, 17-2-94, 17-2-138, 19-1-100, 19-2-98 and 19-2-100.

From 50-100 mg of mudstone was retrieved from each section and oxidized using 30% H_2_O_2_ under low heat for a minimum of an hour, and longer if rock fragments remained mostly intact^23,29^. Samples were rinsed with distilled water a minimum of five times with centrifugation, and the final slurries stored in glass vials at 4 °C. For analysis with scanning electron microscopy (SEM), an aliquot of each slurry was air dried onto a piece of heavy duty aluminum foil, trimmed, and attached to an aluminum stub with apiezon wax. Samples were coated with a mixture of gold and palladium for 2 min with a Polaron Model E sputter coater and observed with a Leo (Zeiss) 982 FESEM, or a FEI Nova NanoSEM 450, field emission scanning electron microscope^23,29^.

Details of the cyst are as described by the guidelines prepared by the International Statospore Working Group^35^. Measurements of the diameters of cysts, pores, collars and spines were made directly from SEM micrographs. Measurements of the pore and collar were made on specimens where these structures were facing straight up. Specimens were not tilted. Spine lengths were estimated from ones along the midsection positioned parallel with the stub surface.

## Results

Large concentrations of an unidentified cyst were uncovered in seven samples from the Giraffe Pipe core, with especially abundant numbers of specimens found in samples from stratum 19-1-100. Many of the cysts from 19-1-100 were unique in that they still contained attached scales, allowing identification as *Mallomonas ampla* Siver & Lott.

### Scales of *Mallomonas ampla* (Fig. 1a–c)

**Figure 1:**
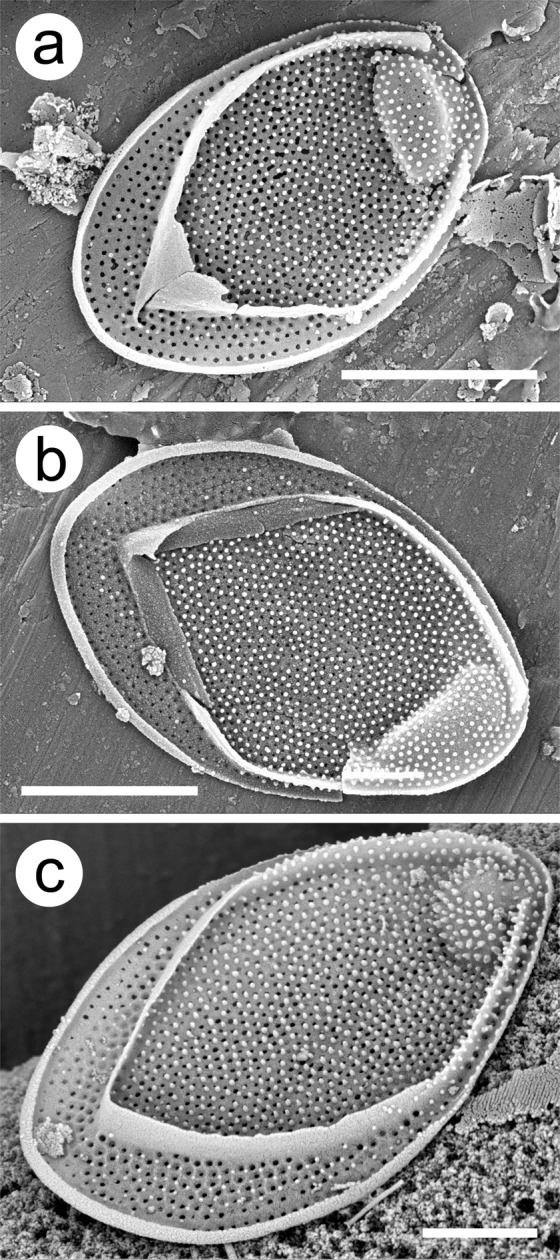
(**a**–**c**) Scanning electron micrographs of isolated scales of *Mallomonas ampla* from the Giraffe Pipe locality. Note the concentric rows of base plate pores on the posterior flange, the surface papillae especially concentrated on the shield and dome, the thin V-rib which is continuous with the anterior submarginal ribs, and the wide, shallow and slightly retracted dome. Scale bar = 2 (a,b) and 1 (c) µm.

Scales of *M*. *ampla* are oval with a perforated base plate, posterior rim, V-rib and a shallow dome structure (Fig. 1a–c). Scales are large and can range upwards in length to 7 µm^23^. The posterior rim, arms of the V-rib, and anterior submarginal ribs are all thinly constructed. The posterior rim is especially shallow and typically extends slightly further along one margin. Base plate pores cover the entire scale, are more or less evenly spaced, and form distinctive concentric rows in the posterior flange. The base of the V-rib can be strongly hooded, but is thin, and the V-rib arms are continuous with the anterior submarginal ribs. On scales with broader domes, the anterior submarginal ribs are often extended into short wing-like structures. The dome is shallow, usually broadly oval, and set back from the anterior margin. The shield and dome are covered with numerous, small, evenly spaced papillae. The posterior flange may possess scattered papillae, but this portion of the scale generally lacks numerous papillae.

### Cyst of *Mallomonas ampla* (Figs. 2–4)

**Figure 2:**
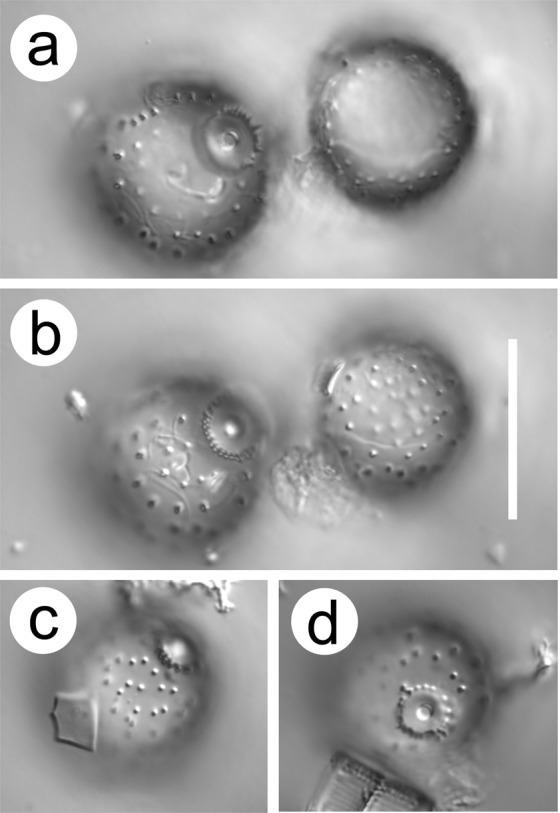
Light micrographs of *Mallomonas ampla* cysts from the Giraffe Pipe locality. (**a**,**b**) Two cysts imaged at different focal planes. The cyst on the left depicts details of the pore-collar complex, while the cyst on the right represents the posterior hemisphere. (**c**,**d**) Cysts depicting the random pattern of spines surrounding the pore-collar complex. Scale bar = 20 µm.

**Cyst GP31** Siver (Fig. 2a)

**Image #:** GP 5020

**Location:** Stratum 19-1-100 of the early Eocene core from the Giraffe Pipe Fossil Locality.

#### Cyst description

**Figure 3:**
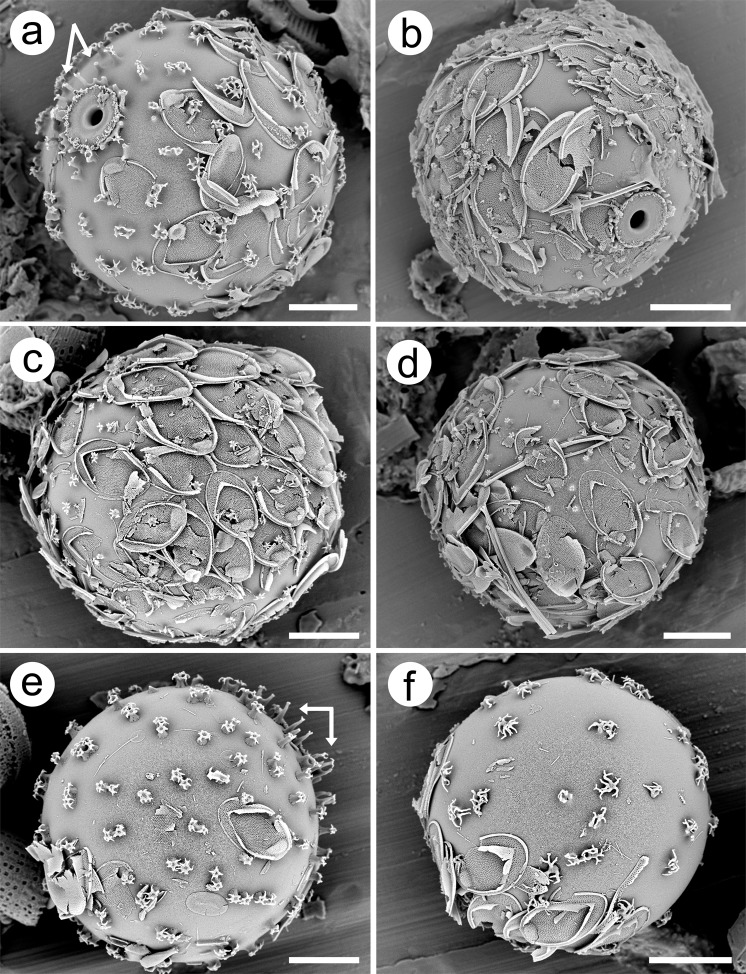
Scanning electron micrographs of *Mallomonas ampla* cysts from the Giraffe Pipe locality with varying degrees of attached scales. (**a**–**b**) Cysts oriented to show the details of the distinctive collar-pore complex. Groups of splayed spines and longer spines lacking splayed apices (arrows) are especially evident on (a). (**c**,**d**) Cysts with numerous attached scales, still displaying their original positions within the cell covering on (**c**). (**e**,**f**) Cysts with widely and unevenly-spaced spines with splayed apices, many of which are in small groups. Longer spines lacking highly splayed apices (arrows, **e**), and shorter spines with highly splayed tips (**f**) are illustrated. Scale bars = 5 µm.

**Figure 4:**
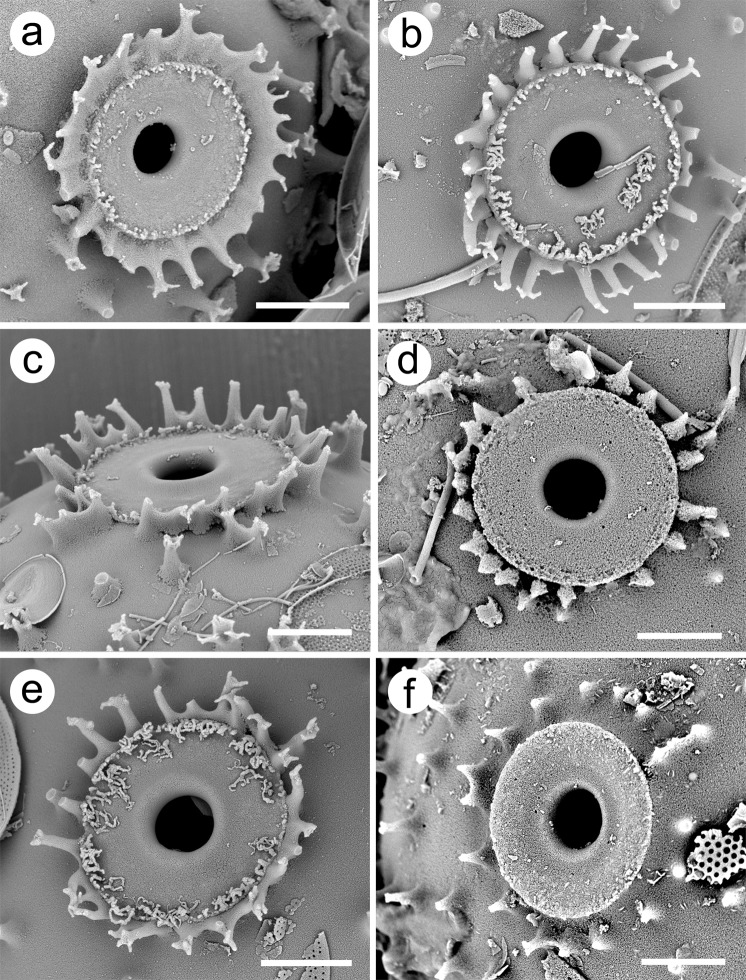
Scanning electron micrographs illustrating the collar-pore complex of *Mallomonas ampla* cysts from the Giraffe Pipe locality. The pore is surrounded by a wide flat collar, which in turn is surrounded by a ring of spines. The spacing and degree to which the spines are fused varies from highly fused (**a**), to most spines being partially fused near the base (**b**,**c**,**e**), to isolated spines (**d**), to lacking a complete ring (**f**). Scale bars = 2 µm.

Cysts are large, spherical with a mean diameter of 19.9 ± 1.3 µm, covered with spines, and with a wide flattened collar that is surrounded by a ring of spines (Figs. 2–4). With the exception of the spines, the cyst wall is smooth. The majority of spines range in length from 0.5–0.9 µm, have a wide base, decrease in diameter with distance from the cyst wall, and terminate with a splayed apex consisting of a series of finger-like projections that are more or less parallel with the cyst wall (Fig. 3a,e,f). On most spines the splayed apices consist of five to ten projections, each of which may further bifurcate. Although spines are solid, there is a depression on the apical end within the center of the terminal projections. Spines are widely spaced and occur singularly, or often in groups of two and rarely three or four. Spines in the anterior hemisphere in the vicinity of the collar are often longer, more slender, with bifurcate apices; these spines have a mean length of 1 µm and lack heavily splayed tips (arrows on Fig. 3a,e). The pore is small, circular, with slightly conical sides and a mean diameter of 1.3 ± 0.1 µm (Fig. 4a–f). The collar is wide with a mean diameter of 4.5 ± 0.3 µm, circular, apically flattened and fused with the cyst wall (Fig. 4a–f). The outer margin of the collar is slightly thickened and often lined with a series of siliceous nodules of twisted projections (Fig. 4a–f). The collar is, in turn, surrounded by a ring of long slender spines that project slightly away from the collar; most of these spines have bifurcate or trifurcate apices.

#### Variation in cyst characters

**Figure 5:**
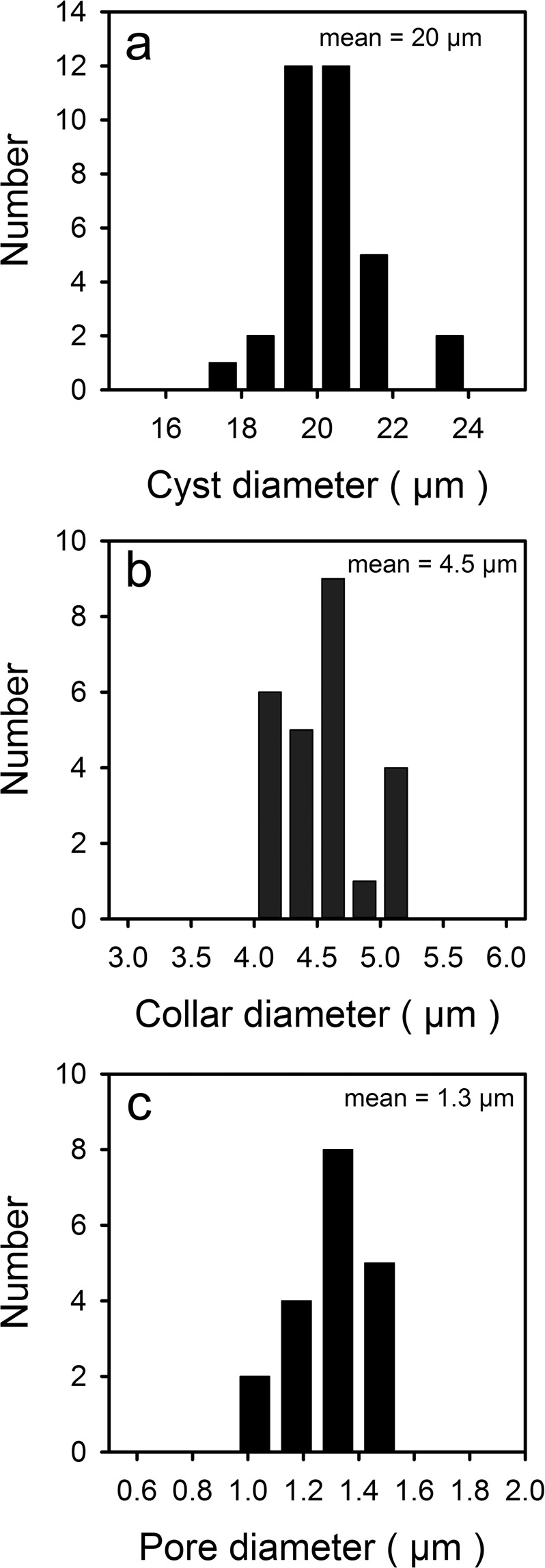
Frequency distributions of **a**) cyst diameter, **b**) collar diameter, and **c**) pore diameter for *Mallomonas ampla* cysts uncovered from the Giraffe Pipe locality.

Although the diameter of cysts ranged from 17–23 µm (n = 34), 29 were between 19–21 µm (Fig. 5a). The diameter of the collar (Fig. 5b) and pore (Fig. 5c) were also relatively stable ranging from 4–5 µm and 1–1.5 µm, respectively. A few cysts had spines with highly splayed and elongated finger projections that often bend downward, sometimes resting on the cyst wall (Fig. 3f). On most cysts, the bases of the spine forming the ring that surrounds the collar were closely spaced and often fused together (Fig. 4a–c,e). Sometimes the spines become fused over half their length, forming what could be considered as a secondary collar (Fig. 4a). On the other end of the spectrum, on a few specimens the spines were fewer and more widely spaced (e.g. Fig. 4d), or not even juxtaposed to the collar itself (e.g. Fig. 4f).

#### Additional observations

**Figure 6:**
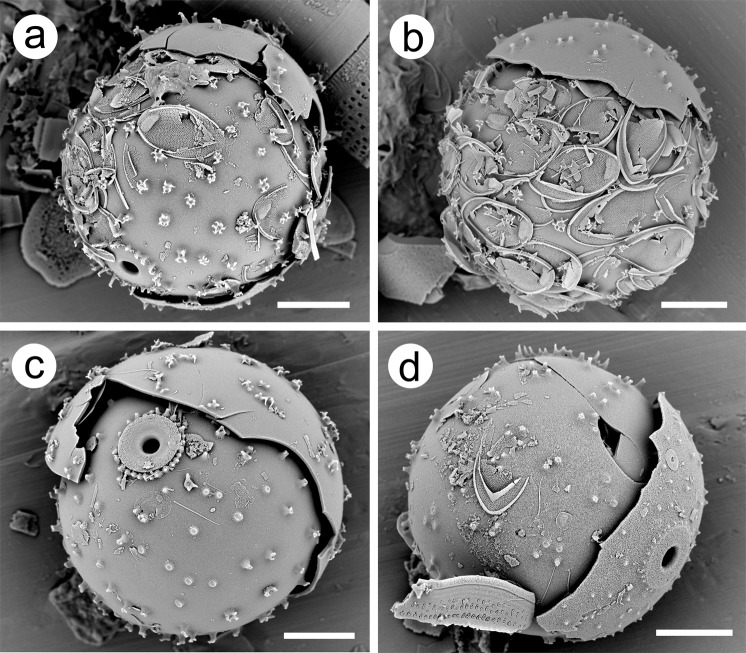
Scanning electron micrographs of *Mallomonas ampla* cysts from the Giraffe Pipe locality that appear to be contained within slightly larger, or “parent,” cysts. Inner cysts in (**a**,**b**,**d**) have attached scales, while no scales are observed on the cyst in (**c**). The broken outer cyst in (**a**) appears to encircle more than 50% of the circumference of the inner cyst, and the one in (**c**) at least 50% of the inner cyst diameter. Scale bars = 5 µm.

Scales from the forming vegetative cell, and in their original orientation, were fused in place onto most cysts. Although ribbed bristles with the unique hooked foot characteristic of *M*. *ampla* were abundant in the rock samples, only broken pieces were found on cysts (Fig. 3d). There were also a number of cysts in cleaned preparations that appeared to be at least partially surrounded by an additional cyst (Fig. 6a–d). That is, a cyst within a cyst. The inner cyst on most, if not all, of these specimens had attached scales, and in all cases both cyst morphotypes represented those of *M*. *ampla*. On some of the specimens, the outer cyst appeared to encircle more than half of the outer circumference of the inner cyst (e.g. Fig. 6a).

#### Museum specimens

The type specimen for *Mallomonas ampla* is from section GP 17-2-94 of the Giraffe Pipe core and deposited at the Canadian Museum of nature (CANA 85744). As a result of the current project, material from the GP 19-1-100 stratum and two permanent glass slide mounts were also deposited at the Canadian Museum of nature (CANA 128453). Each slide has numerous specimens of the cyst for *M*. *ampla*. They also contain specimens of the rare diatom genus *Ambistria* which is detailed in a separate publication^31^.

#### Coexisting organisms

The 19-1-100 stratum from the Giraffe Pipe core contains an extensive diversity of siliceous microfossils. Along with numerous scales and cysts of *M*. *ampla*, this section of the core is also dominated with microfossils of the planktonic diatom, *Aulacoseira giraffensis*^32^. Remains of the synurophytes *Mallomonas insignis* Penard, *M*. *asmundiae* (Wujek & Van der Veer) Nicholls, and *Synura recurvata* Siver & Wolfe are also common, along with a wide diversity of chrysophyte cyst morphotypes. Other diatoms included *Ambistria* spp., *Fragilariforma virescens* (Ralfs) Williams & Round, and *Nupela mutabilis* Siver, Wolfe & Edlund. Scales of the heterotrophic chrysophyte genera *Paraphysomonas* and *Clathromonas* are also abundant, and the stratum contained scales of several heliozoan species, siliceous plates of the testate amoebae genera *Scutiglypha* and *Euglypha*, and a variety of sponge spicules.

## Discussion

Of the vast number of cyst morphotypes described from studies around the world, only 5% or fewer are linked to specific species^6^. This fact severely limits the usefulness of cysts in ecological, biogeographic and paleolimnological investigations. The limited number of cysts that have been successfully linked to specific vegetative states were done so through direct observation of living organisms in field collections, or induced from cultures in laboratory experiments^6^. Cysts uncovered from sediments are no longer associated with the living cells that produced them, making it impossible to assign them directly to specific organisms^8^. No cyst morphotypes described from sediment samples have ever been found still associated with siliceous scales from the cell covering. This fact is what makes the findings reported here for the Giraffe locality truly remarkable.

All samples from the Giraffe locality containing cysts of *Mallomonas ampla* also had isolated scales of this species, but only specimens from the 19-1-100 stratum still had attached scales allowing for a positive identification. This is extraordinary since specimens were initially treated with oxidizing acids in order to remove them from the rock matrix. Even after treatment with acids, scales remained fused to the cysts in their original orientations. This indicates that not only did the cells forming cysts remain intact upon death, but that the scales became fused together and with the cyst wall during the fossilization process. This phenomenon is unique for fossil remains from the Giraffe locality, but it is not unique for *M*. *ampla*. Cysts of other species with intact scales have also been uncovered from the Giraffe locality, indicating that the conditions in the sediments and overlying water column of this maar lake that resulted in the cells remaining intact were unusual, possibly highly anoxic and undisburbed. Scales still attached to the cysts of another species, *Mallomonas aperturae* Siver, were so well preserved that the original arrangement of the scales on the cell covering could be critically examined^36^. The same situation is now documented for *M*. *ampla* from a different section of the extensive core, and in this case with a significantly greater number of specimens.

The specimens of cysts partially surrounded by a second cyst are curious and perplexing. The most logical explanation is that during the fossilization process the inner cysts became compressed against the outer cyst, breaking through the outer cyst, and ending up within the rock matrix appearing to be within the outer cyst. However, in some cases the outer cyst appears to encircle more than half of the diameter of the inner cyst, which could not be explained by this compression hypothesis. In addition, the inner cysts were never found surrounded by ones other than those representing *M*. *ampla*, despite the presence of many other cyst morphotypes in the sample rock. A second explanation is that after formation, what would be the outer cyst “germinated” internally forming a cell with scales, which, in turn, produced the inner cyst. This phenomenon has never been observed and seems unlikely, yet could explain those specimens where more than half of the circumference of the inner cyst is surrounded by the outer cyst. It is also interesting as remarkable that it is to have uncovered cysts with scales still attached in their original overlapping patterns, it is important to note that these “triple” fossil specimens depicting an inner cyst-attached scale coat-outer cyst also remain attached and fused to each other. Does this indicate that the three units were indeed formed together, supporting the internal germination idea? None of these specimens were found attached to a third outermost cyst which would seem likely under the compression hypothesis.

In a recent literature survey that included 1409 records of modern cysts (chrysophytes and synurophytes), Siver^37^ reported a range in diameter from 2–30 µm, with the majority of cyst types between 5–10 µm. This matches nicely cyst diameter estimates of ca. 3–35 μm given by previous researchers^8,13,38^. In addition, the range in diameter for cyst specimens formed by the same species was remarkably small with 85% of the species forming cysts with a range in diameter of approximately 3 µm or less^37^. This was especially true for taxa with scale forming cell coverings. Some of the variation in cyst diameter is likely due to differences in the sizes of vegetative cells at the time of formation^6^. Compared to the results reported by Siver^37^, the cyst formed by *Mallomonas ampla* is on the larger end of the spectrum, and the range in diameter is consistent with the results reported in that study. In addition, the diameters of the collar and of the pore were both remarkably consistent, yielding a mean ratio of 3.5 ± 0.5. The ratio of cyst diameter to pore diameter was large with a mean of 16 ± 0.8. The small size of the pore relative to the cyst is remarkable since when the cyst germinates, the newly formed (and naked) cell must squeeze out of the pore.

Compared to the structure of the collar and pore, there was a wide range of variation in the character of the spines, both within and between specimens. Most of the variation was in the length of the spine and the degree to which the apex was splayed into the finger projections. Shorter spines with more complex apices are the most common morphology and these spines typically cover the majority of the cyst wall. Longer and more slender spines with less splayed apices were restricted to the anterior hemisphere and usually near the collar complex. A hypothesis explaining the variability in spine morphology is that spine length and the degree of lateral splaying of the spine apex for chrysophytes with a siliceous scale covering is dependent on the distance from the developing cyst to the confining cell covering. Formation of spines begins from the cyst wall and progresses outwards. As spines lengthen in an organism with a confining outer scale covering, they come up against the scale coat causing the silica deposition process to bend laterally, resulting in the splayed structure of the apex. Specimens with scales still intact showing splayed apices in contact with the scales clearly supports this hypothesis. Under this hypothesis, the shorter the distance from the cyst wall to the scale covering, the smaller would be the length of the spine. The majority of synurophytes have oval or obovate-shaped cells^1,25,39^. Most cysts are spherical and when they form the anterior hemisphere usually faces the anterior end of the cell^1^. This means that if the cell has an oval shape, that the distance between the developing cyst and scale covering would be greater extending from the anterior hemisphere, and less between the sides and posterior portions of the cyst. Thus, there would be more space for development of longer spines and more complex and projecting collars on the anterior hemisphere, yielding longer spines with no or less splayed apices.

Although the structures of cysts are unknown for many species bearing scale coverings, there is support for the confinement hypothesis resulting in splayed spine apices. At least four other species of *Mallomonas* are known to produce cysts with various types of splayed apices. These include *Mallomonas muskokana* (Nicholls) Siver & Wolfe, *M*. *acaroides* var. *acaroides* Perty emend. Ivanov, *M*. *pseudocoronata* Prescott, and *M*. *crassisquama* (Asmund) Fott. Siver^40^ reported an almost identical spine structure for *M*. *muskokana* as reported here for *M*. *ampla*. Kristiansen^25^ noted the spines of *M*. *acaroides* var. *acaroides* to be “irregularly curved with blunt disc-like tips, which are surrounded by a whorl of short, thin twisted projections”. This describes spines referred to herein as those with splayed apices. Kristiansen^25^ further notes that the cyst formed by *M*. *crassisquama* has spines similar to those of *M*. *acaroides* var. *acaroides*. Spines of *M*. *pseudocoronata* are described as slightly curved with flat splayed tips^25,41^. Interestingly, a similar type of spine spaying is found on the ends of linking spines of some species of the diatom genus *Aulacoseira* (e.g. *A*. *crassipunctata* and *A*. *lirata*^42^). In this case, developing spines become splayed laterally into free spaces as they come in contact with confining silica structures on adjoining valves.

Knowing the morphological structure of *M*. *ampla* cysts could prove useful in discovering the cyst morphotypes for the two closely related species in this lineage, *M*. *multisetigera* and *M*. *neoampla*. Collections containing these species, or surface sediments from waterbodies with active populations, could be examined for cysts with similar spine and collar characters as those for *M*. *ampla*. Although matches made using this technique would only provide indirect evidence, it would nonetheless be worthwhile evidence.

In summary, the numerous specimens of *M*. *ampla* cysts with attached scales from the cell covering in the Giraffe Pipe core are remarkable and further emphasize the uniqueness of this fossil locality. The morphology of the cyst, especially the complex collar, is unique among known cyst types, and the splayed nature of the spine tips is likely a function of forming within a space confined by the scales of the cell covering.

## Data Availability

All data generated for this study is included in this publication. Raw material from stratum 19-1-100 of the Giraffe Pipe core and permanent prepared glass slides with numerous specimens have been deposited at the Canadian Museum of Nature and can be borrowed on request.
